# High-Performance Work Systems and Nurse Role Stress: Serial Indirect Associations of Psychological Capital and Professional Identity

**DOI:** 10.3390/healthcare14101272

**Published:** 2026-05-08

**Authors:** Lu Zhao, Zhengxue Qiao, Jiawei Zhou, Tianyi Bu, Kexin Qiao, Xuan Liu, Yanjie Yang

**Affiliations:** 1 Psychology and Health Management Center, Harbin Medical University, Harbin 150081, China; zhaolu@hrbmu.edu.cn (L.Z.); qiaozhengxue_0@163.com (Z.Q.); 102457@hrbmu.edu.cn (J.Z.); cindybu@hrbmu.edu.cn (T.B.); 102702@hrbmu.edu.cn (K.Q.); 102605@hrbmu.edu.cn (X.L.); 2 Department of Human Resource Management, Health Management College, Harbin Medical University, Harbin 150081, China

**Keywords:** high-performance work systems, role stress, nurses, psychological capital, professional identity

## Abstract

**Background**: Role stress is common among frontline nurses and is associated with demanding work conditions and organizational contexts. High-performance work systems (HPWSs) represent bundled human resource management practices that may be relevant to nurse role stress. This study examined the association between perceived HPWSs and role stress and further evaluated the possible roles of psychological capital and professional identity in this relationship. **Methods**: A multicenter cross-sectional survey was conducted from May to October 2024 in 10 tertiary public hospitals across four Chinese cities. Frontline registered nurses were recruited using cluster sampling and completed an anonymous questionnaire assessing perceived HPWSs, role stress, psychological capital, and professional identity. Descriptive statistics, univariate analyses, and Pearson correlation analyses were performed. Indirect associations were tested using PROCESS with 5000 bootstrap resamples. Sensitivity analyses were conducted with additional covariate adjustment and after excluding the role clarity items from the HPWS measure. **Results**: A total of 2824 valid questionnaires were included. The mean role stress score was 2.88 ± 0.69. Perceived HPWS was negatively associated with role stress (*B* = −0.143, *p* < 0.001) and positively associated with psychological capital (*B* = 0.217, *p* < 0.001) and professional identity (*B* = 0.044, *p* = 0.028). Psychological capital was positively associated with professional identity (*B* = 0.191, *p* < 0.001). Bootstrap analyses showed significant indirect associations through psychological capital (effect = −0.075, 95% *CI* [−0.094, −0.056]), professional identity (effect = −0.008, 95% *CI* [−0.015, −0.001]), and their serial linkage (effect = −0.007, 95% *CI* [−0.011, −0.005]). The overall pattern remained broadly similar in the sensitivity analyses. **Conclusions**: Perceived HPWS was associated with lower nurse role stress, with significant indirect association patterns involving psychological capital and professional identity. These findings highlight the potential relevance of both individual psychological resources and organizational human resource practices in understanding nurse role stress.

## 1. Introduction

Nurses are a key professional group within the healthcare system [1]. Compared with many other occupations, nurses in clinical settings are often required to manage multiple demands at the same time, including patient care, interprofessional collaboration, organizational regulations, performance requirements, and career development [2]. As a result, they are more likely to experience role conflict, role ambiguity, and role overload during role enactment, which may contribute to higher levels of role stress [3]. Previous studies have shown that nurse role stress is closely associated with burnout, emotional exhaustion, reduced job satisfaction, stronger turnover intention, and impaired quality of care [4]. It has therefore become an important issue in nursing management and hospital human resource management. In addition, demographic and work-related characteristics may also shape how nurses perceive and respond to role demands [5,6,7]. This suggests that nurse role stress does not arise from a single source, but reflects the combined influence of individual characteristics and workplace conditions.

Existing research on nurse role stress has mainly focused on stress measurement, group differences, and adverse outcomes [8]. Its underlying formation mechanisms remain insufficiently explained. The Job Demands Resources theory suggests that nursing is characterized by high job demands and substantial emotional demands [9]. Insufficient resources may intensify stress experiences in this context. Role stress theory proposes that stress arises when role demands do not match an individual’s abilities, available resources, or role cognition. When nurses face conflicting role expectations, unclear role boundaries, or excessive task demands, they are more likely to experience role stress [10]. Conservation of Resources theory further suggests that external resources may facilitate the accumulation of internal resources and shape the way individuals respond to stress. Taken together, integrating organizational and individual resources may provide a more comprehensive understanding of nurse role stress [11].

High-performance work systems (HPWS) refer to a set of human resource management practices designed to enhance employees’ abilities, motivation, and opportunities for participation [12]. These practices typically include training and development, performance feedback, employee involvement, and incentive support [13]. Unlike broader constructs such as general organizational support or routine managerial practices, HPWS emphasize a more integrated system of formal human resource practices. When nurses perceive that their hospitals provide clear career pathways, fair performance evaluation, continuous training support, and adequate incentives, they are more likely to develop a stronger sense of control and support in the workplace. This may reduce uncertainty and burden during role enactment. In occupations characterized by high demands, the adequacy of organizational resources is often closely related to employees’ stress experiences [14]. Role stress theory also suggests that clear and supportive institutional environments are commonly associated with lower levels of mismatch between role demands and resource availability [15]. Previous studies have shown that supportive human resource practices are generally associated with higher work engagement, stronger organizational commitment, and lower levels of occupational stress [16,17]. However, direct and systematic evidence on the relationship between HPWS and role stress remains limited in nurse populations. Based on these considerations, the following hypothesis was proposed.

**H1.**  *HPWS are negatively associated with nurse role stress.*

However, the relationship between organizational resources and stress outcomes may not be entirely direct. It may also operate through individuals’ internal psychological resources. Psychological capital is generally defined as a positive psychological state that develops during personal growth and coping [18]. It mainly includes four dimensions: self efficacy, hope, resilience, and optimism [19]. Previous research has shown that psychological capital is often associated with lower emotional exhaustion, better work adaptation, and more favorable mental health [20]. This suggests that it may serve as an important protective factor in nurses’ responses to occupational stress [21]. At the same time, organizational conditions such as training support, opportunities for participation, and positive incentives may also be linked to higher levels of psychological capital among nurses [22]. Access to external resources may be related not only to stress experiences, but also to the accumulation of internal resources [23]. Therefore, psychological capital may represent an important pathway linking HPWS and nurse role stress. Based on these considerations, the following hypothesis was proposed.

**H2.**  *Psychological capital mediates the association between HPWS and nurse role stress.*

Professional identity refers to an individual’s sense of value, meaning, and emotional attachment to their professional role [24]. It is an important psychological foundation for professional socialization and occupational adaptation. Higher levels of professional identity usually reflect a stronger sense of professional belonging, greater occupational mission, and higher role acceptance [25,26]. This may help nurses interpret role demands in nursing work in a more positive way and may reduce stress related to role conflict, role ambiguity, and role overload to some extent. Previous studies have found that nurses with higher professional identity often show greater occupational stability, higher work engagement, and more positive role understanding [27]. In contrast, those with lower professional identity are more likely to experience imbalance and stress in high intensity work settings [28]. At the same time, organizational environments characterized by fairness, developmental support, and supportive practices may strengthen nurses’ positive evaluations of both their profession and their workplace, thereby reinforcing professional identity [29]. Accordingly, professional identity may represent another important pathway linking HPWS and nurse role stress. Based on these considerations, the following hypothesis was proposed.

**H3.**  *Professional identity mediates the association between HPWS and nurse role stress.*

Psychological capital and professional identity may not operate independently. Nurses with higher psychological capital are generally more likely to maintain positive emotional experiences, stronger self efficacy, and better psychological resilience [30,31]. They may also be more likely to perceive meaning, value, and a sense of belonging in their professional roles. COR theory suggests that resource acquisition and accumulation are often progressive. External resources may facilitate the accumulation of internal resources, and internal psychological resources may further shape how individuals understand and engage with their professional roles [32]. In this context, the organizational support reflected in high performance work systems may first be associated with the development of nurses’ positive psychological resources. On this basis, nurses may be more likely to interpret the professional meaning and value of nursing work in a positive manner, which may in turn be associated with stronger professional identity. Higher professional identity may then be related to lower levels of role stress. Although previous studies have separately examined the roles of high-performance work systems, psychological capital, and professional identity in occupational adaptation, few studies in nurse populations have incorporated psychological capital and professional identity into the same model and examined their serial mediating roles in the association between HPWS and role stress. Accordingly, the following hypothesis was proposed.

**H4.**  *Psychological capital and professional identity serially mediate the association between HPWS and nurse role stress.*

**Figure 1:**
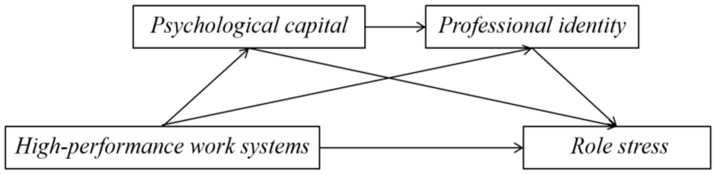
Hypothesized serial mediation model.

Therefore, this study aimed to examine the association between HPWS and nurse role stress. It also sought to test the possible association of psychological capital and professional identity in this relationship. The hypothesized model is presented in Figure 1. This study may contribute to a better understanding of the formation of nurse role stress and may provide evidence to inform organizational and psychological support in nursing management.

## 2. Methods

### 2.1. Study Design and Participants

This multicenter cross-sectional study was conducted between May and October 2024. A multistage cluster-based sampling strategy was adopted to recruit registered nurses in frontline clinical positions from 10 public tertiary hospitals across four Chinese cities, namely Hangzhou, Qingdao, Tianjin, and Guangzhou. First, the four cities were selected as study sites based on regional coverage and study feasibility. Second, tertiary hospitals within these cities were used as the sampling frame, and 10 hospitals were randomly selected in total. Third, within each participating hospital, eligible frontline nurses were selected using random sampling, and 300 questionnaires were distributed at each hospital. The project was approved by the Ethics Committee of the Harbin Medical University (HMUIRB2022042) and was conducted in accordance with the Declaration of Helsinki.

The inclusion criteria were as follows: (1) holding a valid nurse practice certificate and being officially registered; (2) having worked in a frontline clinical position in the current hospital for at least one year, to ensure relatively stable exposure to routine departmental management and organizational practices; and (3) providing written or electronic informed consent after receiving full study information. The exclusion criteria were as follows: (1) holding a nursing department management position; (2) being in the internship, advanced training, probation, or standardized training stages; (3) being continuously absent from clinical duties for more than one month during the survey period because of sick leave, maternity leave, overseas assignment, or similar reasons; and (4) receiving systematic psychiatric or psychological treatment at the time of the survey.

Sample size was estimated using G*Power 3.1 for multiple linear regression. Based on an F test, a small effect size (f^2^ = 0.02), a significance level of 0.05, a statistical power of 0.90, and 12 predictors, the minimum required sample size was 902. After increasing this number by 20% to account for invalid questionnaires or missing data, the target sample size was at least 1083 [33]. A total of 3000 questionnaires were distributed across the 10 hospitals. During data cleaning, 176 questionnaires were excluded, including 64 with completion times below the predefined threshold, 85 with logical inconsistencies, and 27 with substantial missingness in key variables. Ultimately, 2824 valid questionnaires were included in the final analyses, corresponding to an effective response rate of 94.13%. Hospital-specific numbers of distributed, excluded, and included questionnaires are presented in Supplementary Table S1.

### 2.2. Measures

#### 2.2.1. General Information Form

A researcher-developed general information form was used to collect demographic and work-related characteristics based on previous literature and the aims of this study. The form included sex, age, marital status, educational level, professional title, years of work experience, monthly income, weekly working hours and the number of night shifts in the previous month.

#### 2.2.2. Role Stress

Role stress was measured using the Chinese version of a 13-item scale assessing role conflict, role ambiguity, and role overload, originally derived from Peterson et al. [34] and adapted by Li and colleagues [35]. The scale contains 3 items for role conflict, 5 for role ambiguity, and 5 for role overload. Each item is rated on a 5-point Likert scale ranging from 1 (strongly disagree) to 5 (strongly agree), yielding a total score range of 13 to 65, with higher scores indicating greater perceived role stress. This scale has been used in Chinese nursing and healthcare-related research [36]. In the present study, Cronbach’s *α* was 0.952. CFA was conducted to examine whether the observed data were consistent with the intended three-factor structure, and the results indicated good model fit (χ^2^/df = 4.370, RMSEA = 0.035, CFI = 0.996, TLI = 0.995). Detailed factor loading results are presented in Supplementary Table S2.

#### 2.2.3. Psychological Capital

Psychological capital was measured using the 24-item Psychological Capital Questionnaire (PCQ) [37]. The scale comprises four dimensions: self-efficacy, hope, resilience, and optimism. Items are rated on a 6-point Likert scale, with total scores ranging from 24 to 144; higher scores indicate greater psychological capital. A representative item is as follows: “When meeting with department leaders, I am very confident in presenting matters within my scope of work.” This scale has been widely used in Chinese occupational and healthcare samples [38]. In the present study, Cronbach’s *α* was 0.960. CFA was performed to test whether the data supported the original four-factor structure, and the fit indices indicated good model fit (χ^2^/df = 4.680, RMSEA = 0.036, CFI = 0.991, TLI = 0.989). Detailed factor loading results are shown in Supplementary Table S3.

#### 2.2.4. High-Performance Work Systems

Perceived HPWS was assessed using a 31-item scale developed by Mihail [39]. The scale measures nurses’ perceived organizational HRM practice support across seven dimensions: recruitment and selection, training and development, participation in decision-making, employment security, performance management, compensation management, and role clarity. All items are rated on a 5-point Likert scale, with total scores ranging from 31 to 155; higher scores indicate stronger perceived implementation of HPWS. A representative item is as follows: “The hospital evaluates us based on annual assessment goals and organizational requirements and provides feedback on the appraisal results.” In the present study, Cronbach’s *α* was 0.876. CFA was conducted to examine whether the observed data were consistent with the intended seven-factor structure, and the fit indices supported good model fit (χ^2^/df = 4.016, RMSEA = 0.033, CFI = 0.987, TLI = 0.985). Detailed factor loading results are provided in Supplementary Table S4.

#### 2.2.5. Professional Identity

Professional identity was measured using the 30-item scale developed by Liu et al. [40]. It includes five dimensions: professional cognitive evaluation, professional social support, professional social interaction competence, coping with professional frustration, and professional self-reflection. Items are rated on a 5-point Likert scale ranging from 1 (strongly inconsistent) to 5 (strongly consistent), with total scores ranging from 30 to 150; higher scores indicate stronger professional identity. A representative item is as follows: “I believe that those who devote themselves to their profession will gain abundant returns from their career.” This scale has been widely used in Chinese nurse populations [41]. In the present study, Cronbach’s *α* was 0.959. CFA was conducted to test whether the observed data supported the intended five-factor structure, and the model showed good fit (χ^2^/df = 4.610, RMSEA = 0.036, CFI = 0.987, TLI = 0.984). Detailed factor loading results are presented in Supplementary Table S5.

### 2.3. Data Collection and Quality Control

Coordinators at each hospital received standardized training before the survey. Data were collected anonymously through the Wenjuanxing online platform. IP address restrictions were applied to reduce duplicate submissions, and a minimum completion time threshold of 15 min was set in advance. Following data collection, questionnaires were screened using predefined quality control criteria. Questionnaires were excluded if they showed (1) completion times below the prespecified threshold of 15 min; (2) clear logical inconsistencies; or (3) more than 20% missingness in key study variables used in the main analyses. Only questionnaires that satisfied the quality control criteria were retained for the final analytic dataset.

### 2.4. Statistical Analysis

Statistical analyses were performed using SPSS 27.0, AMOS 24.0, and Hayes’ PROCESS macro (Version 4.0). Descriptive statistics were used to summarize sample characteristics and study variables. Group differences were examined using independent-samples *t* tests or one-way ANOVA, and Pearson correlation analyses were performed to assess associations among continuous variables. Internal consistency was evaluated using Cronbach’s *α* coefficients, and CFA was conducted to examine whether the observed data were consistent with the originally proposed factor structures. Common method bias was assessed using Harman’s single-factor test. Multicollinearity was assessed using variance inflation factors (VIFs). Missing data were handled through case-wise exclusion according to predefined quality control criteria, and no imputation was performed. Mediation analyses were conducted using PROCESS Model 6 with 5000 bootstrap resamples. Educational level, professional title, monthly income, weekly working hours, and number of night shifts in the previous month were included as covariates based on prior literature and conceptual relevance. Indirect effects were considered statistically significant when the 95% bootstrap confidence interval did not include zero. Sensitivity analyses were conducted to examine the robustness of the findings, including additional adjustment for all measured demographic and work-related variables and estimation of the models after excluding the role clarity items from the HPWS score. All tests were two-sided, and statistical significance was set at *p* < 0.05.

## 3. Results

### 3.1. Sample Characteristics and Univariate Analyses

This study included 2824 nurses, of whom 92.03% were female. Most participants were aged 25–45 years (73.05%), and most were married (66.61%). Educational attainment was mainly a bachelor’s degree (51.45%) or junior college (31.13%). Most participants had a junior professional title (41.15%) or no professional title (29.64%). In terms of work characteristics, 49.40% reported weekly working hours of >44 h. The most common number of night shifts in the previous month was 5–8 (36.97%), followed by 0–4 (27.87%). Univariate analyses showed significant differences in role stress across educational level, professional title, monthly income, weekly working hours, and number of night shifts in the previous month (all *p* < 0.05). For night-shift frequency, the 5–8 night-shift group had a lower mean role stress score than the 0–4 group, whereas the 9–12 and >12 groups had higher mean scores. No significant differences were found for gender, age, marital status, or years of work experience (all *p* > 0.05). Details are presented in Table 1.

### 3.2. Common Method Bias

Harman’s single-factor test extracted 23 factors with eigenvalues greater than 1, accounting for 80.87% of the total variance. The first factor accounted for 17.41% of the variance, suggesting no evidence of severe common method bias based on this preliminary test.

### 3.3. Correlation Analysis

Table 2 shows that perceived HPWS was positively correlated with professional identity (*r* = 0.144, *p* < 0.01) and psychological capital (*r* = 0.512, *p* < 0.01), and negatively correlated with role stress (*r* = −0.263, *p* < 0.01). Professional identity was positively correlated with psychological capital (*r* = 0.210, *p* < 0.01) and negatively correlated with role stress (*r* = −0.203, *p* < 0.01). Psychological capital was also negatively correlated with role stress (*r* = −0.338, *p* < 0.01).

### 3.4. Mediation Analyses

Before the mediation analyses, multicollinearity diagnostics indicated no serious collinearity among the predictors (maximum VIF = 1.405; all tolerance values >0.1; Durbin–Watson = 1.912). To examine the indirect associations between HPWS and role stress, mediation analyses were conducted using PROCESS Model 6. Indirect effects were estimated using 5000 bootstrap resamples with 95% confidence intervals (CIs). Educational level, professional title, monthly income, weekly working hours, and number of night shifts in the previous month were included as covariates based on prior literature and conceptual relevance. Table 3 presents the regression results for the serial mediation model. Perceived HPWS was positively associated with psychological capital (*B* = 0.217, *t* = 19.235, *p* < 0.001) and professional identity (*B* = 0.044, *t* = 2.204, *p* < 0.05). Psychological capital was positively associated with professional identity (*B* = 0.191, *t* = 6.118, *p* < 0.001). In the final model predicting role stress, perceived HPWS (*B* = −0.143, *t* = −5.319, *p* < 0.001), psychological capital (*B* = −0.347, *t* = −8.146, *p* < 0.001), and professional identity (*B* = −0.179, *t* = −7.036, *p* < 0.001) were all significantly associated with role stress.

Table 4 presents the bootstrap estimates of the total, direct, and indirect associations. The total association between HPWS and role stress was significant (effect = −0.234, 95% CI [−0.284, −0.183]), and the direct association remained significant after the mediators were included (effect = −0.143, 95% CI [−0.196, −0.090]). Significant indirect associations were observed through psychological capital (effect = −0.075, 95% CI [−0.094, −0.056]) and professional identity (effect = −0.008, 95% CI [−0.015, −0.001]). A significant serial indirect association through psychological capital followed by professional identity was also observed (effect = −0.007, 95% CI [−0.011, −0.005]). These findings support the presence of indirect associations between HPWS and role stress through psychological capital, professional identity, and their sequential pathway.

### 3.5. Sensitivity Analysis

Sensitivity analyses were conducted to examine the robustness of the main findings. When the serial mediation model was estimated with additional adjustment for all measured demographic and work-related covariates, the direction, statistical significance, and overall pattern of the key associations remained broadly unchanged. When the model was estimated using a modified HPWS score that excluded the role clarity items, the overall pattern of associations also remained similar. Taken together, these supplementary analyses support the robustness of the main findings. Detailed results are presented in Supplementary Tables S6 and S7.

## 4. Discussion

This study examined the association between HPWS and nurse role stress and further tested the possible roles of psychological capital and professional identity in this relationship. HPWS were negatively associated with role stress, and this association remained significant after psychological capital and professional identity were included in the model. Significant indirect associations were observed through psychological capital, through professional identity, and through their serial linkage.

In this sample, the overall level of nurse role stress was moderate, which is broadly consistent with the high-intensity and rapidly changing nature of clinical nursing work and the sustained coordination required in everyday practice [42]. Nurses often need to respond simultaneously to expectations related to care quality and safety, efficiency and time constraints, and ongoing emotional labor and communication demands. These features are commonly associated with higher perceived role conflict, role ambiguity, and role overload [43]. Accordingly, nurse role stress may be understood as an occupational health concern shaped by both work structure and organizational support [44].

Univariate analyses showed significant differences in role stress across educational level, professional title, monthly income, weekly working hours, and the number of night shifts in the previous month. These findings suggest that variation in role stress may be more closely related to work arrangement and organizational context than to basic demographic characteristics alone [45]. In particular, differences across educational level and professional title may be associated with variation in professional competence, role clarity, autonomy, and access to organizational resources, all of which may relate to how nurses perceive and manage role demands [46,47]. Differences across income may also be linked to perceived reward, fairness, and organizational support [48]. Role stress also differed across weekly working-hour categories, with higher levels generally observed in nurses with longer working hours. This pattern is consistent with the possibility that greater time demands may increase perceived role burden [49]. In addition, the differences in role stress across night-shift schedules did not follow a linear pattern. A moderate level of night-shift schedules may reflect a more regular and predictable scheduling arrangement, which may facilitate adaptation to work rhythms and role expectations. By contrast, heavier night-shift schedules may increase fatigue, disrupt recovery, and intensify role burden. At the same time, a very low frequency of night shifts may reflect less stable scheduling arrangements or a lower degree of adaptation to night work [50]. These differences may help contextualize the observed association between HPWS and nurse role stress from an organizational perspective.

The negative association between HPWS and nurse role stress remained significant after psychological capital and professional identity were included in the model, suggesting that this relationship was not fully accounted for by individual resource factors alone [51]. In nursing settings characterized by high demands, HPWS may be understood as a bundle of organizational practices related to training, feedback, participation, and support. Unlike broader constructs such as general organizational support or routine managerial practices, HPWS emphasize a more integrated system of formal human resource practices. These organizational features may be relevant to how nurses perceive their work environment and available resources, and may therefore be associated with lower role stress [52].

The indirect association through psychological capital suggests that HPWS were associated with higher psychological capital, which was further associated with lower role stress. Psychological capital reflects positive psychological resources such as self-efficacy, hope, resilience, and optimism [53]. These resources are commonly linked to stronger coping capacity and better psychological adjustment under demanding work conditions [54]. In organizational contexts characterized by stronger HRM support, nurses may be more likely to report greater psychological capital, and this pattern is broadly consistent with lower perceived role stress [55].

The indirect association through professional identity indicates that higher perceived HPWS was associated with stronger professional identity, which was in turn associated with lower role stress. Professional identity reflects nurses’ sense of value, belonging, and internalization of their professional role [56,57]. Organizational environments characterized by transparent evaluation, developmental support, and participatory practices may be more likely to reinforce such professional perceptions [58]. In this context, stronger professional identity may be associated with more adaptive appraisal of role demands and lower perceived stress.

A serial indirect association from HPWS to role stress through psychological capital and then professional identity was also observed. This pattern suggests that organizational support may first be reflected in nurses’ positive psychological resources and may then relate to how they understand and value their professional role [59,60]. When psychological resource reserves are more stable, nurses may be more likely to develop consistent value affirmation and internalization of professional roles through ongoing workplace interactions and feedback, which is consistent with higher levels of professional identity [61]. Compared with examining psychological capital or professional identity separately, this sequential pattern provides a more integrated view of how organizational and individual resources may be linked to role stress in nursing contexts [62]. These findings support the relevance of considering both psychological and professional processes when interpreting the association between HPWS and nurse role stress.

## 5. Theoretical and Practical Implications

The present findings have both theoretical and practical implications. This study extends research on nurse role stress by showing that it may be linked not only to individual factors, but also to organizational conditions. The results suggest that HPWS may be associated with nurse role stress both directly and indirectly through psychological capital and professional identity. This supports a more integrated perspective that connects organizational resources, psychological resources, and professional meaning. It also contributes to current discussions on mental health in nursing by highlighting the relevance of organizationally oriented and resource-based strategies.

The findings suggest that reducing nurse role stress may require attention not only to individual support, but also to broader management practices. Clearer role expectations, stronger training support, fairer feedback, participatory management, and more consistent incentives may help create more supportive work environments. At the same time, interventions to support psychological capital and strengthen professional identity may also be relevant. The observed differences across working hours and night-shift frequency also highlight the relevance of scheduling burden in nursing management, particularly with regard to recovery opportunities and fairness in work arrangement.

## 6. Strengths, Limitations, and Future Directions

This study has several strengths. It included a relatively large sample drawn from multiple tertiary public hospitals, which improves the stability of the findings within comparable hospital settings. It also examined HPWS, psychological capital, professional identity, and nurse role stress within a unified framework, allowing direct and potential indirect associations to be evaluated within the same model. In addition, supplementary analyses with additional covariate adjustment showed broadly similar patterns.

Several limitations should be acknowledged. The cross-sectional design does not permit conclusions about temporal ordering or causality. All variables were measured by self-report, so same-source bias, recall bias, and measurement error cannot be excluded. Participants were recruited from tertiary public hospitals in four cities, which limits generalizability to other hospital tiers, ownership types, and regional contexts. The analyses were conducted at the individual level, and potential clustering by hospital or unit was not fully modeled. Residual confounding also remains possible, despite additional covariate adjustment. In addition, because the HPWS measure included a role clarity dimension, some conceptual overlap with role stress cannot be completely ruled out. Although supplementary analyses excluding the role clarity items showed broadly similar patterns, this issue should still be considered when interpreting the magnitude of the observed association.

Future research should use longitudinal designs to clarify temporal ordering and should incorporate multi-source data to reduce same-source bias. Studies in more diverse institutional and regional settings are also needed to evaluate the broader applicability of these findings. Multilevel approaches may further help capture the influence of unit-level and hospital-level organizational contexts on nurse role stress.

## 7. Conclusions

Perceived HPWS was associated with lower nurse role stress, with significant indirect association patterns involving psychological capital and professional identity. These findings suggest that nurse role stress may be better understood within a broader organizational and resource-based framework rather than only as an individual stress response. This study therefore contributes to current discussions on mental health in nursing and highlights the potential relevance of systematic human resource management practices in fostering more supportive nursing work environments.

## Figures and Tables

**Table 1:** Univariate associations of sociodemographic and work-related characteristics with role stress.

Variable	Category	n (%)	Role Stress, Mean ± SD	*t*/*F*	*p*
Sex	Male	225 (7.97)	2.84 ± 0.72	−0.76	0.446
	Female	2599 (92.03)	2.88 ± 0.70		
Age (years)	<25	438 (15.51)	2.88 ± 0.71	0.83	0.509
	25–35	1239 (43.87)	2.88 ± 0.71		
	36–45	824 (29.18)	2.88 ± 0.70		
	46–55	271 (9.60)	2.82 ± 0.62		
	>55	52 (1.84)	3.00 ± 0.73		
Marital status	Married	1881 (66.61)	2.88 ± 0.70	2.10	0.099
	Unmarried	782 (27.69)	2.86 ± 0.70		
	Widowed	43 (1.52)	2.72 ± 0.87		
	Divorced	118 (4.18)	3.00 ± 0.70		
Education level	Secondary vocational school or below	230 (8.14)	2.88 ± 0.72	9.29	<0.001
	Junior college	879 (31.13)	2.92 ± 0.70		
	Bachelor’s degree	1453 (51.45)	2.88 ± 0.69		
	Postgraduate degree	262 (9.28)	2.67 ± 0.68		
Professional title	No professional title	837 (29.64)	2.89 ± 0.70	82.83	<0.001
	Junior nurse	1162 (41.15)	3.09 ± 0.69		
	Supervisor nurse	491 (17.39)	2.59 ± 0.63		
	Associate chief nurse	289 (10.23)	2.51 ± 0.47		
	Chief nurse	45 (1.59)	2.36 ± 0.38		
Years of work experience	≤5	1163 (41.18)	2.89 ± 0.69	0.57	0.634
	6–10	866 (30.67)	2.86 ± 0.71		
	11–15	495 (17.53)	2.86 ± 0.68		
	>15	300 (10.62)	2.85 ± 0.74		
Monthly income (CNY)	≤5000	502 (17.78)	2.85 ± 0.67	2.79	0.039
	5001–10,000	1426 (50.50)	2.87 ± 0.70		
	10,001–15,000	775 (27.44)	2.88 ± 0.70		
	>15,000	121 (4.28)	3.05 ± 0.75		
Weekly working hours (h)	<40	376 (13.31)	2.79 ± 0.78	24.86	<0.001
	40	541 (19.16)	2.78 ± 0.74		
	41–44	512 (18.13)	2.73 ± 0.75		
	>44	1395 (49.40)	2.99 ± 0.61		
Number of night shifts in the previous month	≤4	787 (27.87)	2.70 ± 0.67	319.18	<0.001
	5–8	1044 (36.97)	2.56 ± 0.61		
	9–12	628 (22.24)	3.25 ± 0.62		
	>12	365 (12.92)	3.49 ± 0.40		

**Table 2:** Correlations among study variables.

Variables	1	2	3	4
HPWS	1			
PIN	0.144 **	1		
PCQ	0.512 **	0.210 **	1	
RS	−0.263 **	−0.203 **	−0.338 **	1

Note: HPWS, high-performance work system; PIN, professional identity; PCQ, psychological capital; RS, role stress. ** *p* < 0.01.

**Table 3:** Regression results for the serial mediation model.

Predictor	Outcome: PCQ		Outcome: PIN		Outcome: RS	
	*B* (*SE*)	*t*	*B* (*SE*)	*t*	*B* (*SE*)	*t*
Educational level	0.009 (0.007)	0.111	0.009 (0.012)	0.816	−0.040 (0.015)	−2.577 *
Professional title	0.007 (0.005)	1.302	0.025 (0.009)	2.794	−0.121 (0.012)	−10.081 **
Weekly working hours	−0.003 (0.004)	−0.559	−0.005 (0.008)	−0.648	0.062 (0.011)	5.755 **
Monthly income	0.353 (0.007)	49.865 **	−0.007 (0.016)	−0.418	0.064 (0.022)	2.928 *
Number of night shifts in the previous month	0.347 (0.008)	42.611 **	−0.037 (0.017)	−2.144 *	−0.013 (0.023)	−0.562
HPWS	0.217 (0.011)	19.235 **	0.044 (0.019)	2.204 *	−0.143 (0.027)	−5.319 **
PCQ			0.191 (0.031)	6.118 **	−0.347 (0.043)	−8.146 **
PIN					−0.179 (0.025)	−7.036 **
*R*	0.865		0.225		0.435	
*R* ^2^	0.748		0.051		0.189	
*F*	*F* = 1395.731, *p* < 0.001		*F* = 21.414, *p* < 0.001		*F* = 82.074, *p* < 0.001	

Note: HPWS, high-performance work system; PIN, professional identity; PCQ, psychological capital; RS, role stress. SE, standard error. * *p* < 0.05, ** *p* < 0.001.

**Table 4:** Bootstrap direct and indirect mediating effects.

Paths	Effect	Boot SE	Boot LLCI	Boot ULCI	Effect Ratio (%)
Total effect	−0.234	0.025	−0.284	−0.183	-
Direct effect	−0.143	0.027	−0.196	−0.090	61.11
Indirect effect	−0.091	0.011	−0.111	−0.069	38.89
HPWS → PCQ → RS	−0.075	0.010	−0.094	−0.056	32.05
HPWS → PIN → RS	−0.008	0.004	−0.015	−0.001	3.42
HPWS → PCQ → PIN → RS	−0.007	0.002	−0.011	−0.005	2.99

Note. Boot LLCI: lower limit of the 95% confidence interval from bootstrap sampling. Boot ULCI: upper limit of the 95% confidence interval from bootstrap sampling. Bootstrap method: percentile bootstrap.

## Data Availability

The datasets generated and/or analyzed during the current study are not publicly available due to ethical and privacy restrictions but are available from the corresponding author on reasonable request and with appropriate institutional permission.

## Supplementary Materials

The following supporting information can be downloaded at: https://www.mdpi.com/article/10.3390/healthcare14101272/s1, Table S1. Numbers of distributed, excluded, and included questionnaires by participating hospital. Table S2. Standardized factor loadings for the Chinese version of the Role Stress Scale. Table S3. Standardized factor loadings for the Psychological Capital Questionnaire. Table S4. Standardized factor loadings for the perceived High-Performance Work Systems Scale. Table S5. Standardized factor loadings for the Professional Identity Scale. Table S6. Regression results for the serial mediation model with additional covariate adjustment. Table S7. Regression results for the serial mediation model using the modified HPWS score excluding role clarity items.
