# Supplementary material for: High-Performance Work Systems and Nurse Role Stress: Serial Indirect Associations of Psychological Capital and Professional Identity
Source: Healthcare (Basel). 2026 May 8;14(10):1272. doi: 10.3390/healthcare14101272 (PMC13205375; doi:10.3390/healthcare14101272)
Supplement: Supplementary file 1 [file healthcare-14-01272-s001.zip › healthcare-4230327-supplementary.pdf]

Supplementary Table S1. Numbers of distributed, excluded, and included questionnaires by participating hospital

Hospital	Distributed questionnaires	Excluded due to short completion time	Excluded due to logical inconsistencies	Excluded due to missing key variables	Total excluded	Included in final analysis (Effective response ratio, %)
Hospital 1	300	3	12	1	16	284 (94.67)
Hospital 2	300	4	7	2	13	287 (95.67)
Hospital 3	300	5	5	1	11	289 (96.33)
Hospital 4	300	9	6	2	17	283 (94.33)
Hospital 5	300	3	17	3	23	277 (92.33)
Hospital 6	300	4	6	4	14	286 (95.33)
Hospital 7	300	12	5	8	25	275 (91.67)
Hospital 8	300	4	10	3	17	283 (94.33)
Hospital 9	300	6	9	1	16	284 (94.67)
Hospital 10	300	14	8	2	24	276 (92.00)
**Total**	**3000**	64	85	27	176	2824(94.13)

Supplementary Table S2. Standardized factor loadings for the Chinese version of the Role Stress Scale

Item	Role overload	Role ambiguity	Role conflict
RSS1			0.902
RSS2			0.917
RSS3			0.906
RSS4		0.842	
RSS5		0.858	
RSS6		0.878	
RSS7		0.872	
RSS8		0.845	
RSS9	0.882		
RSS10	0.902		
RSS11	0.907		
RSS12	0.913		
RSS13	0.886		

Supplementary Table S3. Standardized factor loadings for the Psychological Capital Questionnaire

Item	Optimism	Self-efficacy	Resilience	Hope
PCQ1		0.838		
PCQ2		0.871		
PCQ3		0.889		
PCQ4		0.895		
PCQ5		0.883		
PCQ6		0.857		
PCQ7				0.758
PCQ8				0.787
PCQ9				0.791
PCQ10				0.817
PCQ11				0.820
PCQ12				0.784
PCQ13			0.810	
PCQ14			0.835	
PCQ15			0.855	
PCQ16			0.861	
PCQ17			0.851	
PCQ18			0.822	
PCQ19	0.867			
PCQ20	0.890			
PCQ21	0.905			
PCQ22	0.906			
PCQ23	0.900			
PCQ24	0.884			

Supplementary Table S4. Standardized factor loadings for the perceived High-Performance Work Systems Scale

Item	Training and development	Performance management	Recruitment and selection	Participation in decision-making	Employment security	Role clarity	Compensation management
HPWS1			0.950				
HPWS2			0.965				
HPWS3			0.961				
HPWS4			0.939				
HPWS5			0.857				
HPWS6	0.867						
HPWS7	0.861						
HPWS8	0.869						
HPWS9	0.860						
HPWS10	0.808						
HPWS11				0.793			
HPWS12				0.823			
HPWS13				0.847			
HPWS14		0.827					
HPWS15					0.782		
HPWS16					0.804		
HPWS17					0.818		
HPWS18					0.811		
HPWS19		0.829					
HPWS20		0.852					
HPWS21		0.872					
HPWS22		0.885					
HPWS23		0.833					
HPWS24							0.900
HPWS25							0.917
HPWS26							0.885
HPWS27						0.955	
HPWS28						0.966	
HPWS29						0.945	

Supplementary Table S5. Standardized factor loadings for the Professional Identity Scale

Item	Professional cognitive evaluation	Coping with professional frustration	Professional social interaction competence	Professional social support	Professional self-reflection
PIN1	0.856				
PIN2	0.869				
PIN3	0.876				
PIN4	0.878				
PIN5	0.869				
PIN6	0.857				
PIN7	0.840				
PIN8	0.826				
PIN9	0.784				
PIN10				0.774	
PIN11				0.808	
PIN12				0.817	
PIN13				0.834	
PIN14				0.825	
PIN15				0.790	
PIN16			0.760		
PIN17			0.789		
PIN18			0.809		
PIN19			0.822		
PIN20			0.830		
PIN21			0.785		
PIN22		0.770			
PIN23		0.798			
PIN24		0.829			
PIN25		0.853			
PIN26		0.846			
PIN27		0.806			
PIN28					0.774
PIN29					0.841
PIN30					0.828

Supplementary Table S6. Regression results for the serial mediation model with additional covariate adjustment

Predictor	Outcome: PCQ		Outcome: PIN		Outcome: RS	
	B (SE)	***t***	***B (SE)***	***t***	***B (SE)***	***t***
Educational level	0.001 (0.007)	0.129	0.009 (0.012)	0.781	-0.041 (0.016)	-2.600*
Professional title	0.007 (0.005)	1.291	0.025 (0.009)	2.793	-0.121 (0.012)	-10.077**
Weekly working hours	-0.003 (0.005)	-0.540	-0.005 (0.008)	-0.684	0.062 (0.011)	5.712**
Monthly income	0.353 (0.007)	49.834**	-0.007 (0.016)	-0.432	0.064 (0.022)	2.935*
Number of night shifts in the previous month	0.348 (0.008)	42.574**	-0.038 (0.017)	-2.160*	-0.013 (0.024)	-0.552
Sex	0.008 (0.019)	0.389	-0.035 (0.032)	-1.082	0.017 (0.044)	0.377
Age	-0.008 (0.005)	-1.510	0.002 (0.010)	0.241	-0.005 (0.013)	-0.397
Marital status	0.003 (0.008)	0.442	-0.004 (0.014)	-0.294	0.004 (0.018)	0.020
Work Years	0.006 (0.005)	1.213	-0.020 (0.009)	-2.257	-0.012 (0.012)	-0.986
HPWS	0.217 (0.011)	19.274**	0.042 (0.020)	2.135*	-0.144 (0.027)	-5.321**
PCQ			0.193 (0.031)	6.185**	-0.347 (0.043)	-8.123**
PIN					-0.181 (0.027)	-7.058**
R	0.865		0.230		0.435	
R^2	0.749		0.053		0.190	
F	F =837.874，P<0.001		F =14.235，P<0.001		F =54.767，P<0.001	

Note：HPWS, high-performance work systems; PIN, professional identity; PCQ, psychological capital; RS, role stress. SE, standard error. *P<0.05，**P<0.001.

Supplementary Table S7. Regression results for the serial mediation model using the modified HPWS score excluding role clarity items

Predictor	Outcome: PCQ		Outcome: PIN		Outcome: RS	
	B (SE)	***t***	***B (SE)***	***t***	***B (SE)***	***t***
Educational level	0.003 (0.007)	0.472	0.009 (0.012)	0.804	-0.053 (0.034)	-1.541
Professional title	0.010 (0.005)	1.991*	0.024 (0.009)	2.729*	-0.093 (0.026)	-3.541**
Weekly working hours	-0.001 (0.005)	-0.105	-0.005 (0.008)	-0.626	0.044 (0.023)	1.859**
Monthly income	0.345 (0.008)	46.128**	0.001 (0.017)	0.013	0.093 (0.051)	1.814
Number of night shifts in the previous month	0.299 (0.007)	42.950**	-0.021 (0.015)	-1.380	-0.036 (0.046)	-0.784
HPWS	0.145 (0.011)	12.945**	0.047 (0.020)	2.346*	-3.467 (0.059)	-58.123**
PCQ			0.176 (0.032)	5.434**	-0.434 (0.098)	-4.444**
PIN					-0.212 (0.056)	-3.752**
R	0.875		0.223		0.809	
R^2	0.766		0.050		0.655	
F	F =1537.605，P<0.001		F =21.013，P<0.001		F =893.516，P<0.001	

Note：HPWS, high-performance work systems; PIN, professional identity; PCQ, psychological capital; RS, role stress. SE, standard error. *P<0.05，**P<0.001.
